# Steroidal Saponins from the Rhizomes of *Anemarrhena asphodeloides*

**DOI:** 10.3390/molecules21081075

**Published:** 2016-08-17

**Authors:** Bing-You Yang, Jing Zhang, Yan Liu, Hai-Xue Kuang

**Affiliations:** Key Laboratory of Chinese Materia Medica (Ministry of Education), Heilongjiang University of Chinese Medicine, Harbin 150040, China; ybywater@163.com (B.-Y.Y.); zhangjing823@yeah.net (J.Z.); lifeliuyan@163.com (Y.L.)

**Keywords:** Asparagaceae, furostanol saponin, spirostanol saponin, MTT, cytotoxicity

## Abstract

Four new steroid saponins **1**–**4** were isolated from the rhizomes of *Anemarrhena asphodeloides* (Asparagaceae), as well as four known saponins: anemarsaponin B (**5**) timosaponin D (**6**), timosaponin E1 (**7**) anemarsaponin B II (**8**). Their structures were established through UV and NMR as well as MS data. All the compounds were evaluated for cytotoxicity against HepG2 and SGC7901 human cancer lines. Compounds **3** and **7** displayed medium antiproliferative activities on HepG2 and SGC7901 cells, with IC_50_ values of 43.90 and 57.90 μM, respectively.

## 1. Introduction

**Figure 1:**
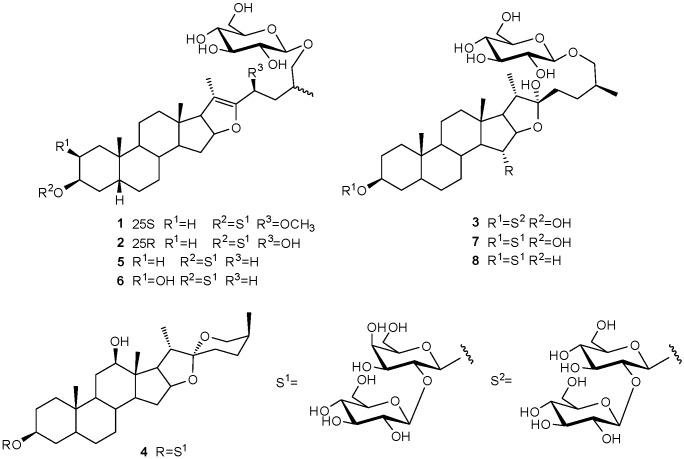
Structures of compounds **1**–**8** from *Anemarrhena asphodeloides*.

The rhizomes of *Anemarrhena asphodeloides* (Asparagaceae) have been used as traditional Chinese medicine for centuries. Phytochemical studies on *Anemarrhena asphodeloides* have led to the identification of a series of compounds, such as steroidal saponins, flavonoids, phenylpropanoids and alkaloids [1]. The most remarkable of the bioactive ingredients are the steroidal saponins. Recently, steroidal saponin studies were focused on a range of bioactivities, such as anti-inflammatory [2], antiplatelet [3,4], and especially anti-tumor properties [5,6]. To explore its active components, we carried out a series of studies on the rhizomes of *Anemarrhena asphodeloides*. Our experiments have led to the isolations of four new steroidal saponins 1–4, which we have named anemarsaponin P–S, along with four known ones, including anemarsaponin B (**5**) [7], timosaponin D (**6**) [8], timosaponin E1 (**7**) [9], anemarsaponin B II (**8**) [10] (Figure 1).

The cytotoxicity of the isolated compounds has also been evaluated by the MTT method. This paper therefore reports the isolation, structural elucidation, and antiproliferative activities of steroidal saponins from *Anemarrhena asphodeloides*.

## 2. Results

### 2.1. Structure Elucidation

**Figure 2:**
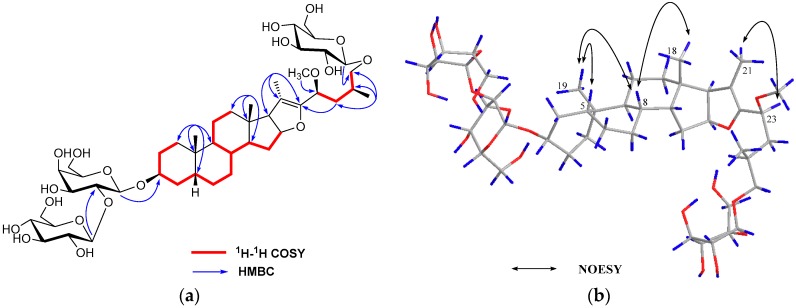
Key HMBC and ^1^H-^1^H COSY correlations (**a**) of compound **1**; Key NOESY correlations (**b**) of compound **1**.

Anemarsaponin P (**1**) was obtained as an amorphous power and its molecular formula was C_46_H_76_O_19_ according to the HR-ESI-MS data (*m*/*z* 955.4644 [M + Na]^+^) (Figure S6). The corresponding ^1^H-NMR (Figure S7) data of the aglycone portion (Table 1) showed four methyl signals at δ_H_ 0.66 (3H, s), 0.99 (3H, s), 1.73 (3H, s) and 1.12 (3H, d, *J* = 6.8 Hz), three anomeric protons at δ_H_ 4.83 (1H, d, *J* = 8.0 Hz), 5.28 (1H, d, *J* = 7.6 Hz) and 4.92 (1H, d, *J* = 8.0 Hz) and a methoxy group at δ_H_ 3.32 (3H, s). The ^13^C-NMR spectrum (Figure S8) showed four methyl groups at δ_C_ 14.6, 24.1, 11.6 and 17.9. Characteristic signals at 109.2 (C-20), 150.4 (C-22) and the secondary carbon signal at δ_C_ 75.5 (C-26) indicated that compound **1** was a Δ^20(22)^-unsaturated furostanol saponin [11]. Comparison of the ^1^H- and ^13^C-NMR spectra in **1** with those of anemarsaponin B (**5**) revealed the ring A–E portions and glycoside moiety of C-3 of the former were consistent with those of **5**. On the other hand, remarkable differences were indicated by the carbon signals from the ring F portion (C-22~C-27). The HMBC correlations between the methoxy signal (δ_H_ 3.32) and C-23 (δ_C_ 73.7) indicated that methoxy group should be placed in C-23, which was proved by the HMBC correlations from H-24_a_ (δ_H_ 2.07) to C-22 (δ_C_ 150.4) (Figure 2a). The key NOESY correlations between H-23 and H-21/H-27 were indicative of α-orientation for H-23 (Figure 2b). Therefore, the methoxy group at C-23 had a β-orientation. On the basis of above features, a 23*S* configuration was established by reference to notation of *R*,*S*-configuration. The absolute configuration of 25*S* in **1** was established by the chemical shift of H_2_-26 (δ_H_ 3.56 and 4.15 ppm, Δδ = 0.59) (Δδ ≥ 0.57 ppm) [11]. Thus, compound **1** was inferred as (23*S*)-3β,26-dihydroxy-23β-methoxyl-5β-furost-20(22)-en. The absolute configurations of the β-glucose and β-galactose [12] were determined as D by GC analysis of their hydrolyzed forms. The linkage of the sugar in **1** was proved by long-range HMBC correlations between δ_H_ 4.83 (H-1′) and δ_C_ 75.5 (C-26, aglycone), δ_H_ 4.92 (H-1′′) and δ_C_ 75.3 (C-3, aglycone) and δ_H_ 5.28 (H-1′′′) and δ_C_ 81.8 (C-2′′, 3-*O*-β-d-galactose) (Figure 2a, Table 2). Thus, the structure of compound **1** was deduced to be (23*S*,25*S*)-26-*O*-β-d-glucopyranosyl-3β,26-dihydroxy-23β-methoxyl-5β-furost-20(22)-en-3β-yl-*O*-β-d-glucopyranosyl-(1→2)-β-d-galactopyranoside.

Anemarsaponin Q (**2**) was an amorphous power and its molecular formula was C_45_H_74_O_19_ according to HR-ESI-MS at *m*/*z* 941.4714 [M + Na]^+^ (Figure S9). The ^13^C-NMR spectrum data (Figure S11) (Table 1) showed four methyl groups at δ_C_ 14.6, 24.0, 11.7 and 17.8. In addition, the carbon signals at δ_C_ 105.1 (C-20), 154.4 (C-22) and the secondary C-26 carbon signal (δ_C_ 75.6) indicated that compound **2** was Δ^20(22)^-unsaturated furostanol saponin [11]. The ^13^C-NMR data of compound **2** was very similar to those of **1**, except for the absence of a methoxy in **2**. Significant differences were observed between **1** and **2** for C-22~C-27. The C-23 that appeared at δ_C_ 73.7 of **1** was instead an upfield-shifted carbon at δ_C_ 63.9 in compound **2**. The above data suggested that a hydroxy group existed at C-23 of **2**, which was supported by the correlations from H-23 to H-24, H-24 to H-25, and H-25 to H-27 in the ^1^H-^1^H COSY spectrum of **2** (Figure S1).

The NOESY cross-peaks between H-23 and H-21 indicated the α-orientation of H-23 and the β-orientation of hydroxyl group for C-23 (Figure S2). Being similar to compound **1**, the absolute configuration of 23S was confirmed by reference to the notation of *R*,*S*-configuration. The 25*R* configuration in **2** was proved by the protons of H_2_-26 (δ_H_ 3.81 and 4.07 ppm, Δδ = 0.26) (Δδ ≤ 0.48 ppm) [11]. Compound **2** afforded d-glucose and d-galactose, identified by GC analysis of their acid hydrolysis derivatives. Compound **2** was thus established as (23*S*,25*R*)-26-*O*-β-d-glucopyranosyl-3β,23β,26-trihydroxy-5β-furost-20(22)-en-3β-yl-*O*-β-d-glucopyranosyl-(1→2)-β-d-galactopyranoside.

Anemarsaponin R (**3**) (Figure S12). had a molecular formula C_45_H_76_O_20_, based on HR-ESI-MS at *m*/*z* 959.4840 [M + Na]^+^. In the ^13^C-NMR spectrum (Figure S14) of **3** (Table 1) four methyl groups (δ_C_ 18.5, 24.7, 16.9 and 17.9) and quaternary carbon (δ_C_ 110.8) suggested that compound **3** was a furostanol saponin [11]. Comparison of ^13^C-NMR data indicated the same skeleton in **3** and timosaponin E1 (**7**). The tiny differences between them were seen in the sugar moiety of C-3. Instead of glucose and galactose in **7**, two glucoses were identified in **3** by the signals at δ_C_ 102.3, 83.6, 78.6, 72.1, 78.7, 63.4 and δ_C_ 106.4, 77.5, 78.4, 72.3, 79.0, 63.2 (Table 2). In addition, the existence of glucose was also confirmed by the coupling constant of 9.6 Hz in H-4 for compound **3** instead of the typical one of galactose [13,14]. Meanwhile, the ^13^C-NMR data of the sugar residue matched those of anemarsaponin C [15]. Their sugar linkages were established by the existence of long-range HMBC correlations between δ_H_ 4.81 (H-1′) and δ_C_ 75.9 (C-26, aglycone), δ_H_ 4.94 (H-1′′) and δ_C_ 75.7 (C-3, aglycone) and δ_H_ 5.39 (H-1′′′) and δ_C_ 83.6 (C-2′′, 3-*O*-β-d-glucose) (Figure S3). The sugars of **3** were also only assignable to d-glucose by GC analysis of their chiral derivatives. The NOESY cross-peaks between the signals of H-23_a_ and H-20, H-15 and H-18 indicated the α-orientations of the hydroxyl groups at C-15 and C-22 (Figure S4). In addition, the NOESY correlations between H-3 and H-1′′, H-5 and H-19 indicated the β-orientations of OH-3, H-5 (Figure S4). Thus, the structure of compound **3** was established as (25*S*)-26-*O*-β-d-glucopyranosyl-3β,15α,22α,26-tetrahydroxy-5β-furost-3β-yl-*O*-β-d-glucopyranosyl-(1→2)-β-d-glucopyranoside.

Anemarsaponin S (**4**) had a molecular formula C_46_H_76_O_19_, on the basis of HR-ESI-MS at *m*/*z* 779.4193 [M + Na]^+^ (Figure S15). In the ^1^H-NMR spectrum (Figure S16) of **4** (Table 1) the signals at δ_H_ 1.08 (3H, s), 1.00 (3H, s), 1.48 (3H, d, *J* = 6.4 Hz) and 1.09 (3H, d, *J* = 6.8 Hz) were assignable to four methyl groups. Correspondingly, the signals at δ_C_ 11.2, 24.0, 14.3 and 16.3 also demonstrated the existences of four methyl groups. In addition, the signal at δ_C_ 110.0 was assignable to the characteristic C-22 carbon signal of a spirostanol saponin. Comparison of the carbon signals revealed multiple similarities between **4** and (25*R*)-5β-spirostane-3β,12β-diol-3-*O*-β-d-glucopyranosyl-(1→2)-β-d-galacto-pyranoside [16], excluding the F ring. In the comparison of the F ring signals (δ_C_ 32.2, 29.6, 31.0, 67.2, 17.7), apparent differences were observed at δ_C_ 26.3, 26.5, 27.6, 65.1, 16.3 of **4**. Meanwhile, the ^13^C-NMR data of F ring in **1** were consistent with markogenin 3-*O*-d-glucopyranosyl-(1→2)-β-d-galactopyranoside [17]. Certainly, both the shift of H_3_-27 (δ_H_ 1.09, d, *J* = 6.8 Hz) and H_2_-26 proton chemistry shift (δ_H_ 3.40 and 4.13) directly confirmed a 25*S* configuration in **4** [18]. Therefore, compound **4** was formulated as (25*S*)-3β,12β-dihydroxy-5β-spirostane-3β-yl-*O*-β-d-glucopyranosyl-(1→2)-β-d-galactopyranoside.

By comparison of NMR data with those reported, the four known compounds were established as anemarsaponin B (**5**) [7], timosaponin D (**6**) [8], timosaponin E1 (**7**) [9], anemarsaponin B II (**8**) [10].

### 2.3. Cytotoxic Activity

**Figure 3:**
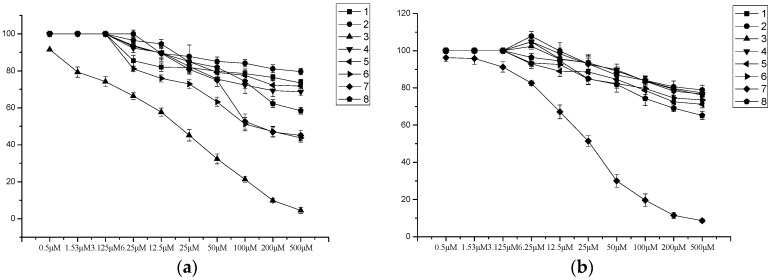
(**a**) Inhibition of HepG2 cell proliferation by the tested compounds; (**b**) Inhibition of SGC7901 cell proliferation by the tested compounds.

Anemarsaponins P-S (compounds **1**–**4**), anemarsaponin B (**5**), timosaponin D (**6**), timosaponin E1 (**7**) and anemarsaponin B II (**8**) were evaluated for their in vitro cytotoxic activities against two human tumor cell lines (HepG2 and SGC7901) through the MTT method. Among them, anemarsaponin R (**3**) showed medium cytotoxicity against HepG2 cells, with an IC_50_ value of 43.90 μM. Timosaponin E1 (**7**) exhibited medium cytotoxicity against SGC7901 cells, with an IC_50_ value of 57.90 μM. The other compounds did not show significant cytotoxicity (IC_50_ > 100 μM) (Table 3). The dose dependent of cell viabilityon HepG2 (**a**) and SGC7901 (**b**) for compounds (Figure 3).

## 3. Experimental Section

### 3.1. General Experimental Procedures

Optical rotations were obtained on a P-2000 polarimeter (JASCO, Tokyo, Japan). IR spectra were obtained on a FTIR-8400S instrument (Shimadzu, Kyoto, Japan). UV spectra were obtained on a Shimadzu UV-1601 instrument. NMR spectra were obtained on DPX 400 NMR instrument (Bruker, Rheinstetten, Germany). HR-ESI-MS were recorded on a Xevo-TOF-MS™ instrument (Waters, Milford, MA, USA). HPLC was performed by using a Waters 515 HPLC system coupled with a Waters 2414 refractive index detector. A Waters XBridge preparative C_18_ column (19 × 250 mm, 10 μm) was used. Macroporous absorption resin D-101 (Cangzhou Bon Adsorber Technology Co., Ltd., Cangzhou, China), MPLC (C-610, Büchi, Flawil Switzerland) and ODS silica gel (YMC Ltd., Kyoto, Japan) were used for column chromatography. MeOH and EtOH were analytical grade and purchased from Tian-Jin Fu Yu Co., Ltd. (Tianjin, China). MeCN was HPLC grade and obtained from J&K Scientific Ltd. (Beijing, China).

### 3.2. Plant Material

The rhizomes of *Anemarrhena asphodeloides* Bunge were collected at Bozhou Country, Anhui Province, People’s Republic of China, in September 2014, and identified by Ruifeng Fan from Heilongjiang University of Chinese Medicine. A voucher specimen (20140930) has been deposited in the laboratory.

### 3.3. Extration and Isolation

The dried slice of rhizomes from *Anemarrhena asphodeloides* (56 kg) were extracted three times with hot water (280 L) under reflux. The solutions were combined and evaporated to give a residue (22.09 kg). The crude was suspended with EtOAc, *n*-BuOH, H_2_O, respectively. The *n*-BuOH elute was concentrated under vacuum to yield the *n*-BuOH–soluble fraction.

A part of the fraction (120 g) was chromatographed by MPLC, eluted with a stepwise gradient of MeOH–H_2_O and finally with MeOH, giving 20 subfractions. Fr.5 (3.1 g) was subjected to ODS column chromatography, eluted with a gradient of MeOH–H_2_O (4:6 to 1:0) to yield Fr.5-1~Fr.5-4. Fr.5-4 (0.8 g) was separated by preparative HPLC (5.0 mL/min, 15% MeCN–H_2_O) and yielded compound **4** (4 mg). Fr.17 (0.2 g) was purified with HPLC (5.0 mL/min, 27% MeCN–H_2_O) to give compound **7** (6 mg). Fr.18 (25 g) was chromatographed over an ODS column to afford Fr.18-1~Fr.18-5. Compound **5** (2.9 g) was crystallized from Fr.18-3. Fr.18-5 was further purified by HPLC (5.0 mL/min, 30% MeCN–H_2_O) to afford compounds **1** (5 mg), **2** (28 mg) and **3** (8 mg). Fr.19 (2 g) was purified by HPLC (5.0 mL/min, 38% MeCN–H_2_O) to give compounds **6** (7 mg) and **8** (57 mg).

Anemarsaponin P (**1**). White amorphous power; ${\left[\alpha \right]}_{D}^{21.7}$−67 (*c* 2.0, MeOH); UV (MeOH) γ_max_ 217 nm; CD Δε +1.5 (231 nm); IR (KBr) ν_max_ 3454, 2930, 1646, 1405, 1070 cm^−1^; ^1^H- and ^13^C-NMR data (Figures S7 and S8), see Table 1 and Table 2; HR-ESI-MS *m*/*z* 955.4644 [M + Na]^+^ (calc. for C_46_H_76_O_19_ 955.4878).

Anemarsaponin Q (**2**). White amorphous power; ${\left[\alpha \right]}_{D}^{23.2}$−7.1 (*c* 1.7, MeOH); UV (MeOH) γ_max_ 211 nm; CD Δε +1.7 (232 nm); IR (KBr) ν_max_ 2418, 2931, 1067, 1033 cm^−1^; ^1^H- and ^13^C-NMR data (Figures S10 and S11), see Table 1 and Table 2; HR-ESI-MS *m*/*z* 941.4714 [M + Na]^+^ (calc. for C_45_H_74_O_19_ 941.4722).

Anemarsaponin R (**3**). White amorphous power; ${\left[\alpha \right]}_{D}^{21.8}$−38.0 (*c* 2.0, MeOH); IR (KBr) ν_max_ 3465, 2935, 1397, 1080 cm^−1^; ^1^Hp and ^13^C-NMR data (Figures S13 and S14), see Table 1 and Table 2; HR-ESI-MS *m*/*z* 959.4840 [M + Na]^+^ (calc. for C_45_H_76_O_20_ 959.4828).

Anemarsaponin S (**4**). White amorphous power; ${\left[\alpha \right]}_{D}^{22}$ −5.0 (*c* 2.0, MeOH); IR (KBr) ν_max_ 3428, 2930, 1397, 1070 cm^−1^; ^1^H- and ^13^C-NMR data (Figures S16 and S17), see Table 1 and Table 2; HR-ESI-MS *m*/*z* 779.4193 [M + H]^+^ (calc. for C_39_H_64_O_14_ 779.4194).

Anemarsaponin B (**5**). White amorphous power; ^1^H-NMR (400 MHz, Pyridine) δ: 0.70 (3H, s, H-18), 1.00 (3H, s, H-19), 1.64 (3H, s, H-21), 1.04 (3H, d, *J* = 6.4 Hz, H-27), 4.95 (1H, d, *J* = 8.0 Hz, H-1′′), 4.85 (1H, d, *J* = 7.6 Hz, H-1′′′), 5.31 (1H, d, *J* = 7.6 Hz, H-1′); ^13^C-NMR (100 MHz, Pyridine) δ: 31.0 (C-1), 27.0 (C-2), 75.3 (C-3), 31.0 (C-4), 37.0 (C-5), 26.9 (C-6), 26.9 (C-7), 35.2 (C-8), 40.2 (C-9), 35.3 (C-10), 21.3 (C-11), 40.1 (C-12), 43.9 (C-13), 54.8 (C-14), 31.4 (C-15), 84.6 (C-16), 64.7 (C-17), 14.4 (C-18), 24.0 (C-19), 103.6 (C-20), 11.9 (C-21), 152.4 (C-22), 34.5 (C-23), 23.7 (C-24), 33.7 (C-25), 75.3 (C-26), 17.2 (C-27), 102.6 (C-1′′), 82.0 (C-2′′), 77.0 (C-3′′), 69.9 (C-4′′), 76.7 (C-5′′), 62.2 (C-6′′), 106.2 (C-1′′′), 75.6 (C-2′′′), 78.1 (C-3′′′), 71.8 (C-4′′′), 78.5 (C-5′′′), 62.8 (C-6′′′), 105.2 (C-1′), 75.3 (C-2′), 78.6 (C-3′), 71.8 (C-4′), 78.7 (C-5′), 62.9 (C-6′).

Timosaponin D (**6**). White amorphous power; ^1^H-NMR (400 MHz, Pyridine) δ: 0.70 (3H, *s*, H-18), 1.00 (3H, s, H-19), 1.64 (3H, s, H-21), 1.05 (3H, d, *J* = 6.4 Hz, H-27), 4.99 (1H, d, *J* = 7.6 Hz, H-1′′), 4.82 (1H, d, *J* = 7.6 Hz, H-1′′′), 5.28 (1H, d, *J* = 7.6 Hz, H-1′); ^13^C-NMR (100 MHz, Pyridine) δ: 40.1 (C-1), 67.2 (C-2), 82.0 (C-3), 31.9 (C-4), 36.6 (C-5), 26.3 (C-6), 26.9 (C-7), 35.3 (C-8), 41.4 (C-9), 37.1 (C-10), 21.5 (C-11), 40.6 (C-12), 43.8 (C-13), 54.7 (C-14), 31.4 (C-15), 84.6 (C-16), 64.7 (C-17), 14.4 (C-18), 23.9 (C-19), 103.4 (C-20), 11.8 (C-21), 152.4 (C-22), 34.4 (C-23), 23.7 (C-24), 33.7 (C-25), 75.3 (C-26), 17.2 (C-27), 103.6 (C-1′′), 81.8 (C-2′′), 75.3 (C-3′′), 69.8 (C-4′′), 77.0 (C-5′′), 62.1 (C-6′′), 106.2 (C-1′′′), 77.0 (C-2′′′), 78.1 (C-3′′′), 71.8 (C-4′′′), 78.6 (C-5′′′), 62.9 (C-6′′′), 105.2 (C-1′), 75.3 (C-2′), 78.6 (C-3′), 71.8 (C-4′), 78.6 (C-5′), 62.9 (C-6′).

Timosaponin E1 (**7**). White amorphous power; ^1^H-NMR (400 MHz, Pyridine) δ: 0.81 (3H, *s*, H-18), 1.00 (3H, s, H-19), 1.28 (3H, d, *J* = 6.8 Hz, H-21), 1.01 (3H, d, *J* = 6.8 Hz, H-27), 4.81 (1H, d, *J* = 7.6 Hz, H-1′′), 4.96 (1H, d, *J* = 7.6 Hz, H-1′′′), 5.26 (1H, d, *J* = 7.6 Hz, H-1′); ^13^C-NMR (100 MHz, Pyridine) δ: 31.0 (C-1), 27.2 (C-2), 75.5 (C-3), 31.0 (C-4), 37.3 (C-5), 27.0 (C-6), 26.5 (C-7), 36.4 (C-8), 40.5 (C-9), 35.4 (C-10), 21.4 (C-11), 41.3 (C-12), 41.6 (C-13), 60.8 (C-14), 79.1 (C-15), 91.4 (C-16), 61.4 (C-17), 18.0 (C-18), 24.1 (C-19), 40.9 (C-20), 16.5 (C-21), 110.4 (C-22), 37.1 (C-23), 28.4 (C-24), 34.5 (C-25), 75.5 (C-26), 17.5 (C-27), 103.3 (C-1′′), 81.8 (C-2′′), 75.3 (C-3′′), 69.9 (C-4′′), 76.8 (C-5′′), 62.1 (C-6′′), 106.2 (C-1′′′), 77.0 (C-2′′′), 78.1 (C-3′′′), 71.7 (C-4′′′), 78.4 (C-5′′′), 62.9 (C-6′′′), 105.2 (C-1′), 75.3 (C-2′), 78.7 (C-3′), 71.8 (C-4′), 78.5 (C-5′), 62.9 (C-6′).

Anemarsaponin BII (**8**). White amorphous power; ^1^H-NMR (400 MHz, Pyridine) δ: 0.81 (3H, *s*, H-18), 0.99 (3H, s, H-19), 1.32 (3H, d, *J* = 6.8 Hz, H-21), 1.02 (3H, d, *J* = 6.8 Hz, H-27), 4.82 (1H, d, *J* = 8.0 Hz, H-1′′), 4.93 (1H, d, *J* = 7.6 Hz, H-1′′′), 5.30 (1H, d, *J* = 7.6 Hz, H-1′); ^13^C-NMR (100 MHz, Pyridine) δ: 31.4 (C-1), 27.5 (C-2), 75.7 (C-3), 31.4 (C-4), 37.4 (C-5), 27.5 (C-6), 27.3 (C-7), 36.0 (C-8), 40.7 (C-9), 35.7 (C-10), 21.6 (C-11), 40.9 (C-12), 41.7 (C-13), 56.9 (C-14), 32.9 (C-15), 81.7 (C-16), 64.5 (C-17), 17.2 (C-18), 24.5 (C-19), 41.1 (C-20), 17.0 (C-21), 111.1 (C-22), 37.6 (C-23), 28.8 (C-24), 34.9 (C-25), 75.9 (C-26), 17.9 (C-27), 103.0 (C-1′′), 82.3 (C-2′′), 77.4 (C-3′′), 70.3 (C-4′′), 77.1 (C-5′′), 63.2 (C-6′′), 106.6 (C-1′′′), 76.0 (C-2′′′), 78.5 (C-3′′′), 72.2 (C-4′′′), 78.9 (C-5′′′), 63.2 (C-6′′′), 105.6 (C-1′), 75.7 (C-2′), 79.1 (C-3′), 72.2 (C-4′), 79.0 (C-5′), 62.6 (C-6′).

### 3.4. Acid Hydrolysis and GC Analysis

The hydrolysis and GC analysis of the four new compounds were carried out for the chiral sugar derivatives. Compounds **1**–**4** (2 mg) were heated with 5 mL 2 M HCl at 90 °C for 3 h. The mixtures were extracted with EtOAc (5 mL) for three times. The sugar residue was dispersed with 1 mL pyridine and reacted with l-cysteine methyl ester hydrochloride (1.5 mg) at 60 °C for 1 h. Then 150 μL of HMDS-TMCS (hexamethyldisilazane–trimethylchlorosilane, 3:1) was added into the mixture that was further reacted at 60 °C for 30 min. The supernatant of the mixture was evaporated to dryness with a N_2_ stream. The residue was separated with H_2_O (0.1 mL) and *n-*hexane (0.1 mL), and the supernatant layer (1 μL) was analyzed by GC. The configurations of the sugar portion for compounds **1**–**4** were determined by comparison the retention times of their derivatives with those of standard d-glucose (*t*_R_ = 15.68 min) and d-galactose (t_R_ = 13.47 min) [19].

### 3.5. Cytotoxic Activity

The isolated compounds were evaluated for their in vitro antiproliferative activities by the MTT method. Doxorubicin was used as positive control (Table 3). Two cell lines, HepG2 and SGC7901, were obtained from the Shanghai Institute of Biochemistry and Cell Biology (Shanghai, China). They were cultured in RPMI 1640 supplemented with 10% FBS, 100 IU/mL penicillin, 100 μg/mL streptomycin in 5% CO_2_ at 37 °C. Cells were cultured in 96-well plates for 24 h with 100 μL complete medium, followed by treating with compounds at different concentrations. 20 μL MTT (5 mg/mL in PBS) was added in the 96-well plates for another 4 h. The solutions were assayed at 490 nm using a VICTOR-X3 ELISA instrument (PerkinElmer, Waltham, MA, USA), after the precipitates were dissolved in DMSO [20]. The cytotoxicities of compounds against HepG2 and SGC7901 were calculated and expressed as IC_50_ values.

## 4. Conclusions

As described in the introduction, in recent years steroidal saponins have become a research hotspot due to their multiple and strong bioactivities, especially their cytotoxic activities against a series of tumor cell lines [5,6,21]. In this paper, four new saponins were isolated from *Anemarrhena asphodeloides* their strictures elucidated, and their antiproliferative activities against HepG2 and SGC7901 were evaluated. Obvious differences were observed between the antiproliferative activities of compounds **3**, **7** and the others. The above results represent a contribution to the discovery of new active ingredients and lead compounds and provide an experimental and scientific basis for drug design and drug discovery.

## Figures and Tables

**Table 1:** ^1^H-NMR and ^13^C-NMR data for aglycone moiety of compounds **1**–**4** (pyridine-*d*_5_).

No.	1		2		3		4	
	δ_H_ mult (*J*, Hz)	δ_C_	δ_H_ mult (*J*, Hz)	δ_C_	δ_H_ mult (*J*, Hz)	δ_C_	δ_H_ mult (*J*, Hz)	δ_C_
**1a**	1.49 m	31.0	1.48 m	31.0	1.48 ^a^	31.6	1.47 ^a^	31.0
**1b**	1.87 ^a^		1.83 ^a^		1.88 ^a^		1.80 ^a^	
**2a**	1.21 ^a^	27.0	1.23 ^a^	27.0	1.45 ^a^	27.3	1.51 ^a^	26.7
**2b**	1.84 ^a^		1.87 ^a^		1.92 ^a^		1.95 m	
**3**	4.28 m	75.3	4.28 m	75.3	4.30 ^a^	75.7	4.31 m	75.5
**4a**	1.45 ^a^	31.0	1.52 m	31.0	1.80 ^a^	31.2	1.81 ^a^	31.0
**4b**	1.81 ^a^		1.89 ^a^		1.82 ^a^		1.85 ^a^	
**5**	2.15 m	37.0	2.18 m	37.0	2.20 m	37.4	2.16 m	36.8
**6a**	0.92 m	26.8	0.98 m	26.9	1.25 ^a^	27.6	1.21 m	27.1
**6b**	1.82 ^a^		1.20 ^a^		1.87 ^a^		1.83 ^a^	
**7a**	1.51 ^a^		1.51 ^a^		1.50 ^a^		0.93 m	
**7b**	1.98 m	26.9	2.00 m	26.9	2.31 m	27.5	1.28 ^a^	26.7
**8**	1.38 ^a^	35.1	1.40 ^a^	35.2	1.86 ^a^	36.9	1.54 ^a^	34.7
**9**	1.22 ^a^	40.2	1.27 ^a^	40.2	1.42 ^a^	40.9	1.46 ^a^	39.4
**10**		35.3		35.3		35.9		35.3
**11a**	1.15 m	21.3	1.15 ^a^	21.3	1.22 ^a^	21.7	1.49 ^a^	31.4
**11b**	1.32 m		1.32 m		1.35 m		1.78 m	
**12a**	1.17 ^a^		1.15 ^a^		1.20 ^a^			
**12b**	1.70 m	40.1	1.72 d (9.2)	40.1	1.68 m	41.6	3.53 dd (4.0, 10.0)	79.5
**13**		44.0		43.9		41.8		46.7
**14**	0.81 m	54.8	0.80 m	54.8	1.53 ^a^	61.4	1.10 m	55.3
**15a**	1.04 ^a^	34.5	1.42 ^a^	34.5	4.40 ^a^	79.5	1.58 m	31.9
**15b**	2.05 m		2.07 dd (5.6, 12.4)				2.09 dd (5.4, 12.0)	
**16**	4.87 m	84.8	4.85 m	84.7	5.05 dd (4, 8.8)	91.9	4.66 t (5.4)	81.4
**17**	2.55 d (10.0)	65.0	2.50 d (10.4)	65.1	2.18 ^a^	61.9	2.22 ^a^	62.8
**18**	0.66 s	14.6	0.70 s	14.6	0.95 s	18.5	1.08 s	11.2
**19**	0.99 s	24.1	0.99 s	24.0	1.04 s	24.7	1.00 s	24.0
**20**		109.2		105.1	2.26 m	41.4	2.18 m	43.6
**21**	1.73 s	11.6	1.75 s	11.7	1.30 d (6.8)	16.9	1.48 d (6.4)	14.3
**22**		150.4		154.4		110.8		110.0
**23**	4.22 m	73.7	4.92 m	63.9	2.09 ^a^ 1.97 m	37.6	2.18 ^a^ 1.36 m	26.3
**24a**	1.87 ^a^	37.3	1.80 br.d (12.4)	39.7	1.70 m	28.9	1.30 m	26.5
**24b**	2.07 ^a^		2.40 m		2.07 ^a^		1.50 ^a^	
**25**	2.21 m	31.1	2.48 br.d (10.4)	31.0	1.*9*2 ^a^	34.9	1.62 ^a^	27.6
**26a**	3.56 m	75.5	3.81 br.d (10.4)	75.6	3.48 dd (6.8, 9.2)	75.9	3.40 br.d (10.8)	65.1
**26b**	4.15 ^a^		4.07 m		4.08 m		4.13 m	
**27**	1.12 d (6.8)	17.9	1.18 d (6.4)	17.8	1.01 d (6.8)	17.9	1.09 d (6.8)	16.3
OCH_3_	3.32 s	56.0						

^a^ Overlapped signals.

**Table 2:** ^1^H-NMR and ^13^C-NMR data for sugar portions of compounds **1**–**4** (pyridine-*d*_5_).

No.	1		2		3		4	
	δ_H_ (mult, *J*, Hz)	δ_C_	δ_H_ (mult, *J*, Hz)	δ_C_	δ_H_ (mult, *J*, Hz)	δ_C_	δ_H_ (mult, *J*, Hz)	δ_C_
Glc-1	4.83 d (8.0)	105.3	4.89 d (7.6)	105.1	4.81 d (7.6)	105.7		
2	4.01 m	75.3	4.07 ^a^	75.2	4.03 ^a^	75.7		
3	3.84 ^a^	78.5	3.97 m	78.6	4.24 br.d (8.8)	79.1		
4	4.20 ^a^	71.7	4.22 t (8.4)	71.8	4.23 br.d (8.8)	72.2		
5	4.21 ^a^	78.6	4.24 m	78.7	3.95 ^a^	78.9		
6	4.36 m 4.45 d (8.0)	62.9	4.39 dd (2.8, 12.4) 4.55 dd (5.6, 12.4)	63.0	4.32 dd (3.2, 14.8) 4.50 br.d (14.8)	63.3		
Gal/Glc-1	4.92 d (8.0)	102.6	4.93 d (7.6)	102.6	4.94 d (7.6)	102.3	4.93 d (8.0)	102.6
2	4.68 t (7.6)	81.8	4.69 t (11.2)	82.0	4.26 m	83.6	4.70 ^a^	81.9
3	4.08 t (7.6)	77.0	4.10 d (11.2)	76.9	4.32^a^	78.6	4.27 dd (3.2, 9.6)	75.3
4	4.57 dd (2.2, 8.4)	69.9	4.58 ^a^	69.9	4.33 br.d (9.6)	72.3	4.59 d (3.2)	69.9
5	4.03 ^a^	76.7	4.04 m	76.6	3.87 m	78.7	4.03 t (8.4)	76.6
6	4.43 br.d (9.2) 4.39 dd (3.6, 9.2)	62.2	4.40 dd (1.8, 12.0) 4.45 br. d (12.0)	62.2	4.40 d (10.4) 4.54 br.d (10.4)	63.4	4.40 dd (1.2, 8.4) 4.45 dd (2.4, 8.4)	62.2
Glc-1	5.28 d (7.6)	106.1	5.30 d (8.0)	106.2	5.39 d (8.0)	106.4	5.30 d (7.6)	106.2
2	4.35 m	75.6	4.31 t (8.4)	75.6	4.09 ^a^	77.5	4.10 m	77.0
3	4.18 ^a^	78.1	4.20 t (8.4)	78.1	4.28 ^a^	78.4	4.22 t (11.2)	78.1
4	4.30 br.d (9.6)	71.8	4.32 m	71.9	4.18 br.d (10.0)	72.1	4.34 t (11.2)	71.8
5	3.84 m	78.5	3.87 m	78.4	4.26 ^a^	79.0	3.86 m	78.5
6	4.53 dd (1.2, 10.8) 4.47 m	62.9	4.36 dd (4.0, 9.6) 4.48 br.d (9.6)	62.9	4.38 d (8.8) 4.57 br.d (8.8)	63.2	4.47 dd (3.6, 8.4) 4.53 dd (1.2, 8.4)	62.8

^a^ Overlapped signals.

**Table 3:** Cytotoxicities of compounds **1**–**8**.

Compounds	IC_50_ (μM)		Compounds	IC_50_ (μM)	
	HepG2	SGC7901		HepG2	SGC7901
**1**	>100	>100	**6**	>100	>100
**2**	>100	>100	**7**	>100	57.90 ± 2.88
**3**	43.90 ± 3.36	>100	**8**	>100	>100
**4**	>100	>100	doxorubicin	8.20 ± 1.25	6.25 ± 2.18
**5**	>100	>100			

## Supplementary Materials

Supplementary materials can be accessed at: http://www.mdpi.com/1420-3049/21/8/1075/s1.

## Abbreviations

The following abbreviations are used in this manuscript:

IR	Infrared
NMR	Nuclear magnetic resonance
UV	Ultraviolet
HR-ESI-MS	High-resolution electrospray ionization mass spectrometry
HMBC	Heteronuclear multiple bond correlation
HSQC	Heteronuclear multiple quantum coherence
NOESY	Nuclear overhauser effect
^1^H-^1^H COSY	Correlation spectroscopy
MeOH	Methanol
HPLC	High performance liquid chromatography
MPLC	Middle pressure liquid chromatography
MeCN	Acetonitrile
GC	Gas chromatography
EtOAc	Ethyl acetate
MTT	3-(4,5-dimethylthiazol-2-yl)-2,5-diphenyl-2-*H*-tetrazolium bromide
