# Supplementary material for: Steroidal Saponins from the Rhizomes of Anemarrhena asphodeloides
Source: Molecules. 2016 Aug 17;21(8):1075. doi: 10.3390/molecules21081075 (PMC6272963; doi:10.3390/molecules21081075)
Supplement: Supplementary file 1 [file molecules-21-01075-s001.pdf]

Supplementary Materials: Steroidal saponins from the Rhizoma of *Anemarrhena asphodeloides*

Bing-You Yang, Jing Zhang, Yan Liu, and Hai-Xue Kuang

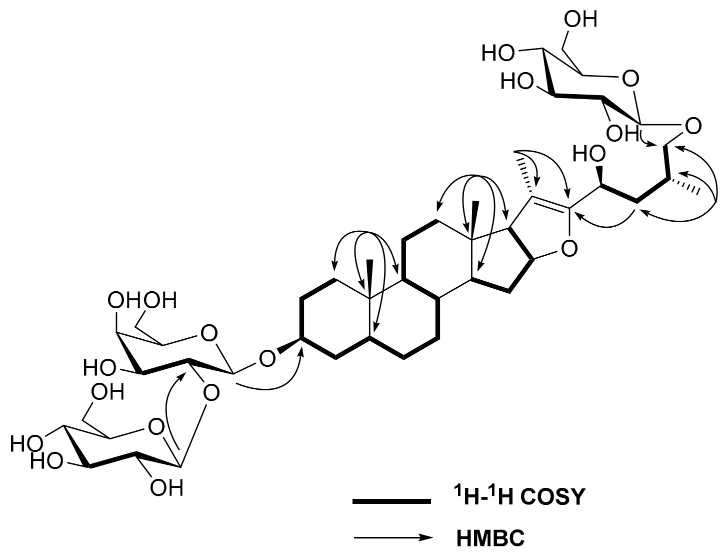

Figure S1. Key HMBC and ^{1}H-^{1}H COSY correlations of compound **2**.

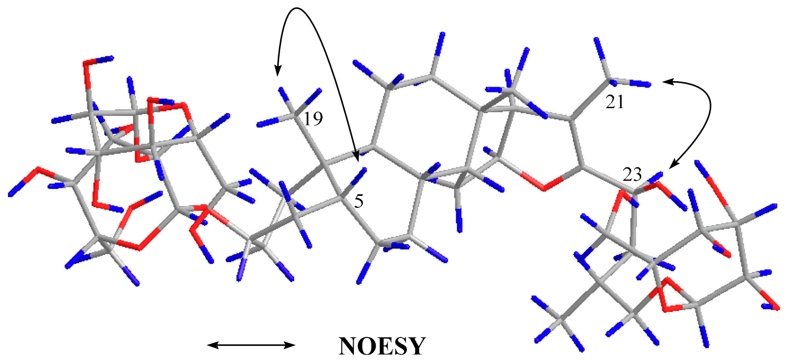

Figure S2. Key NOESY correlations of compound **2**.

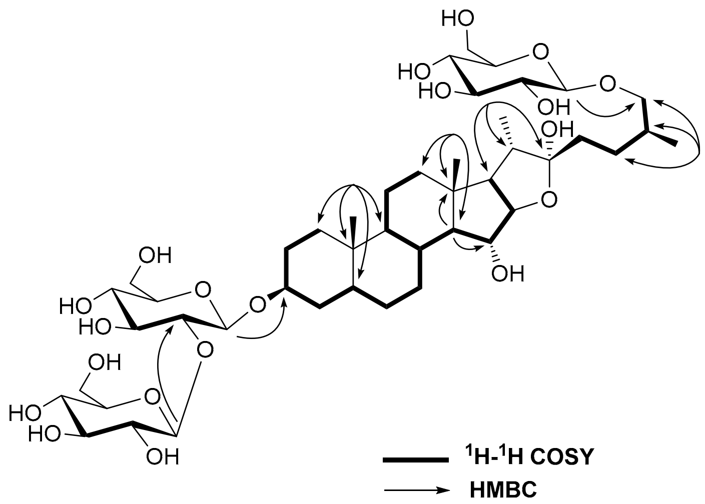

Figure S3. Key HMBC and ^{1}H-^{1}H COSY correlations of compound **3**.

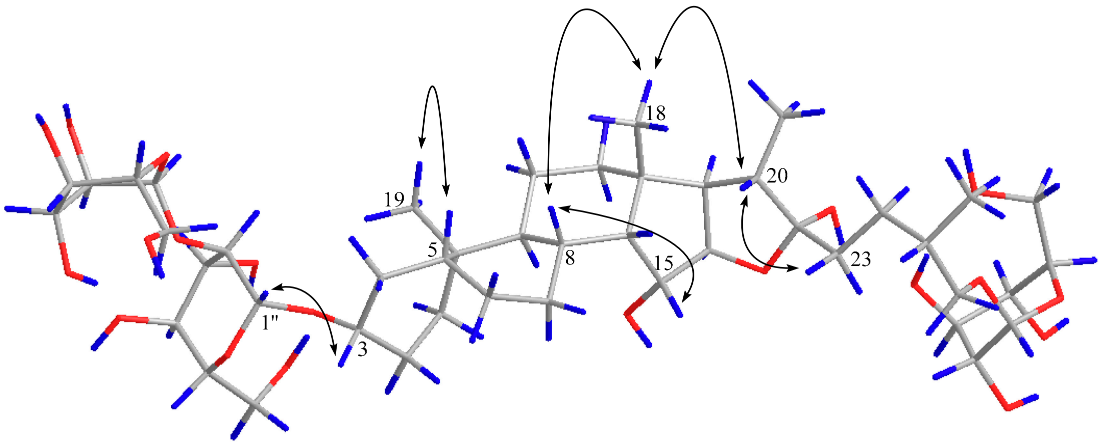

Figure S4. Key NOESY correlations of compound **3**.

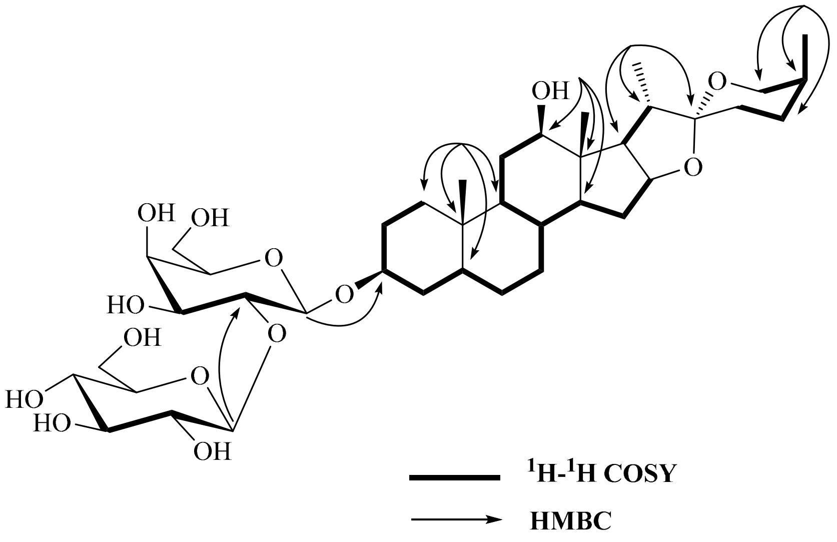

Figure S5. Key HMBC and ^{1}H-^{1}H COSY correlations of compound **4**.

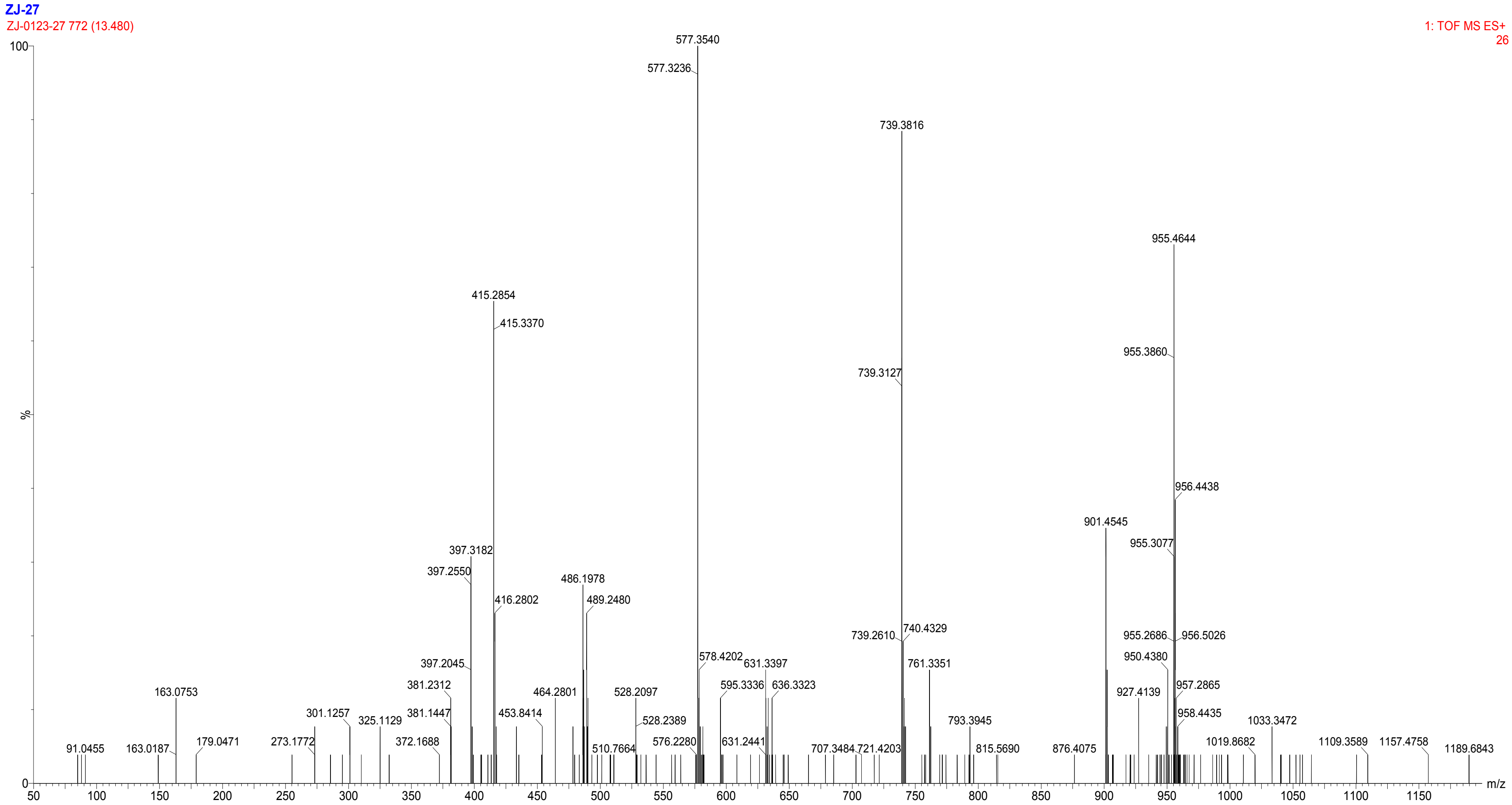

Figure S6. HR-ESI-MS spectrum of compound **1**.

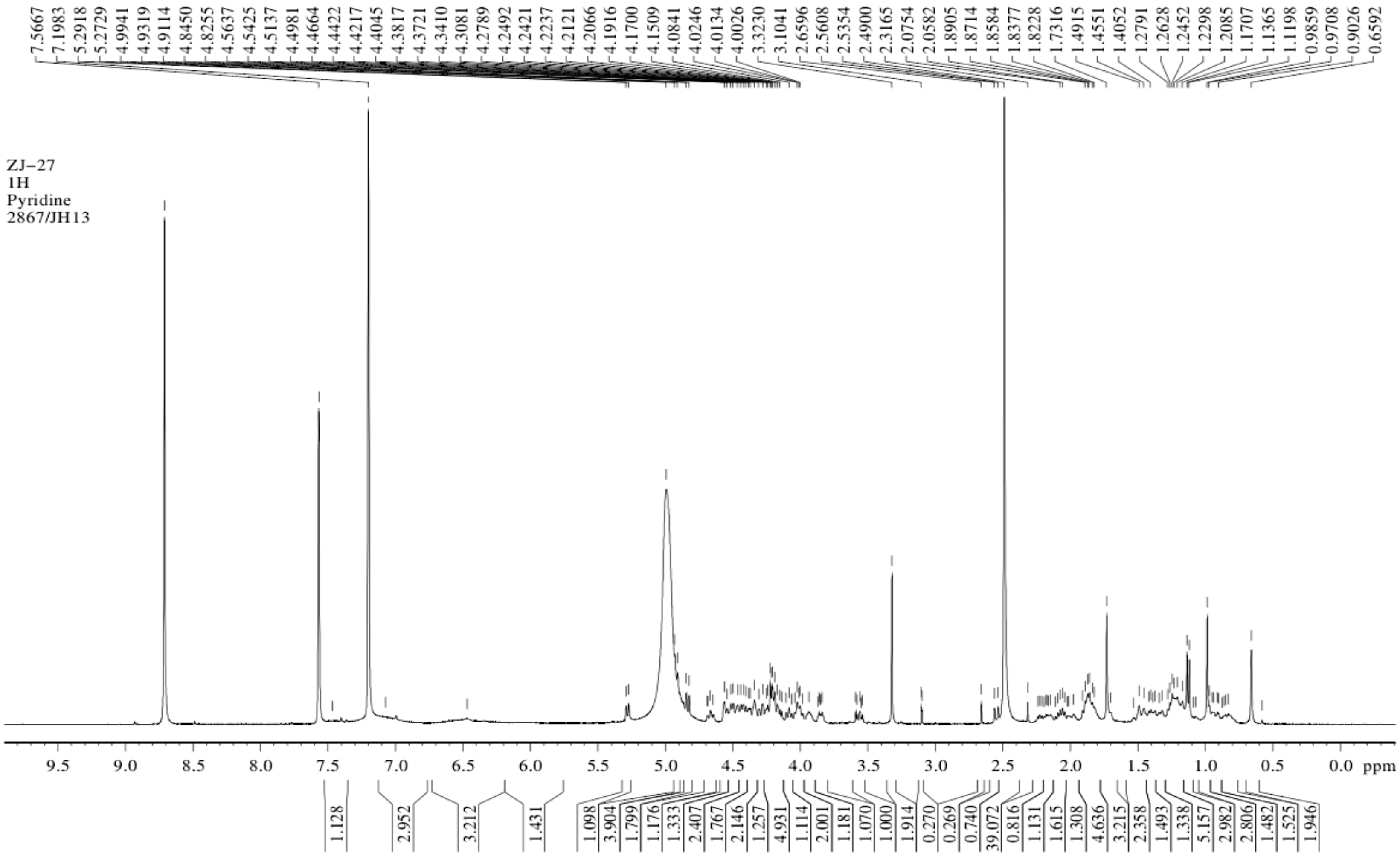

Figure S7. ^{1}H-NMR spectrum of compound **1**.

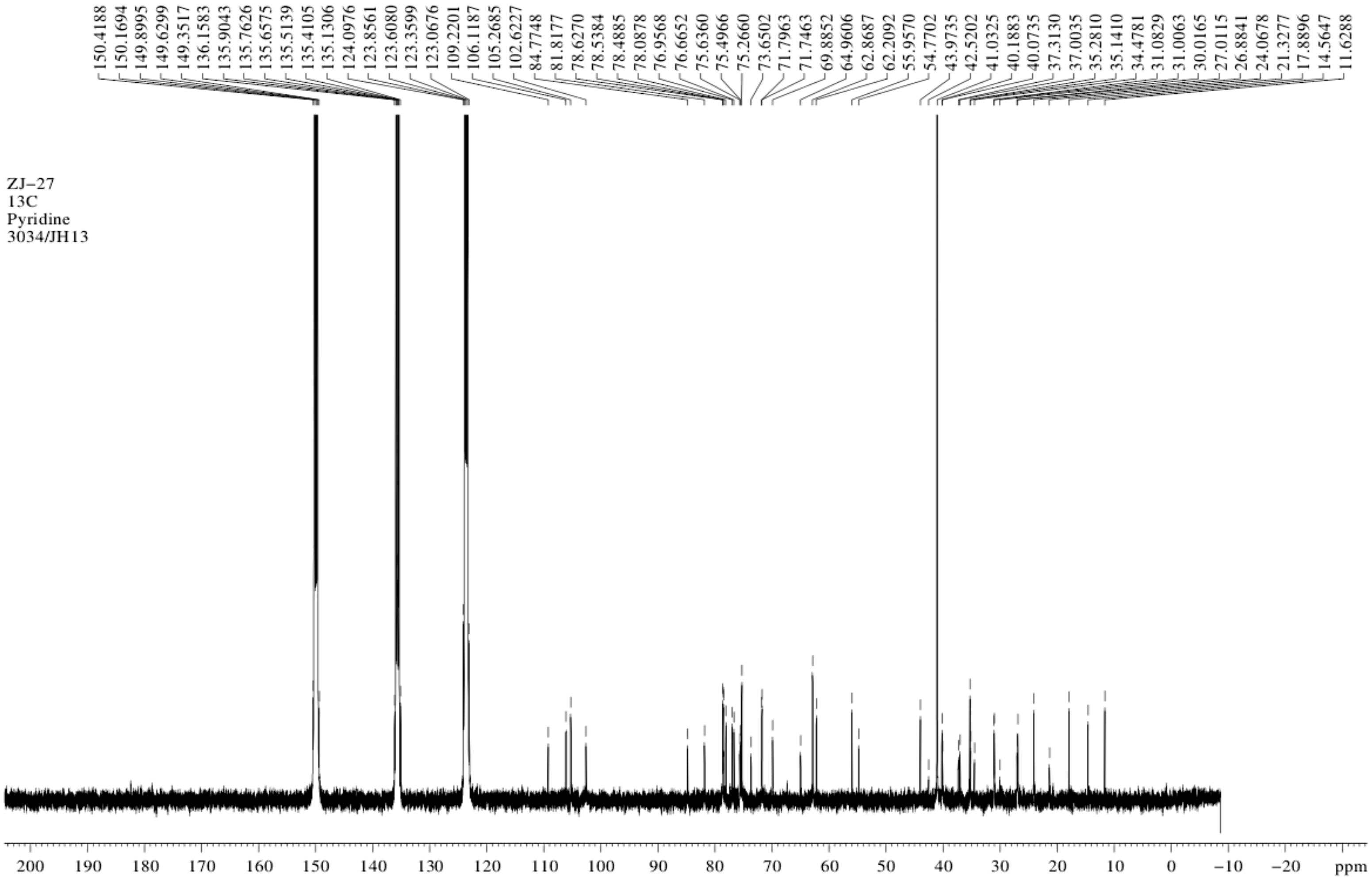

Figure S8. ^{13}C-NMR spectrum of compound **1**.

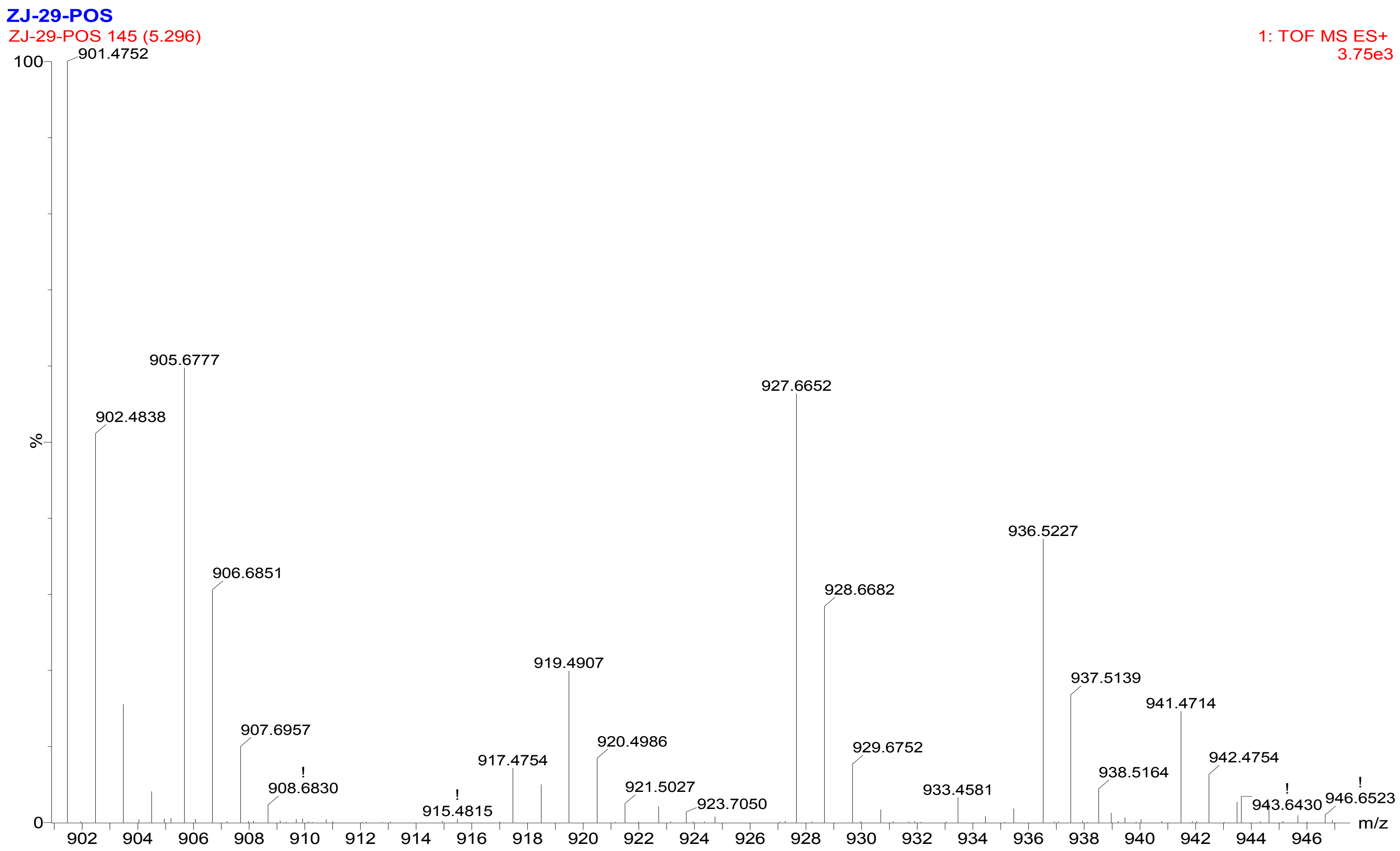

Figure S9. HR-ESI-MS spectrum of compound **2**.

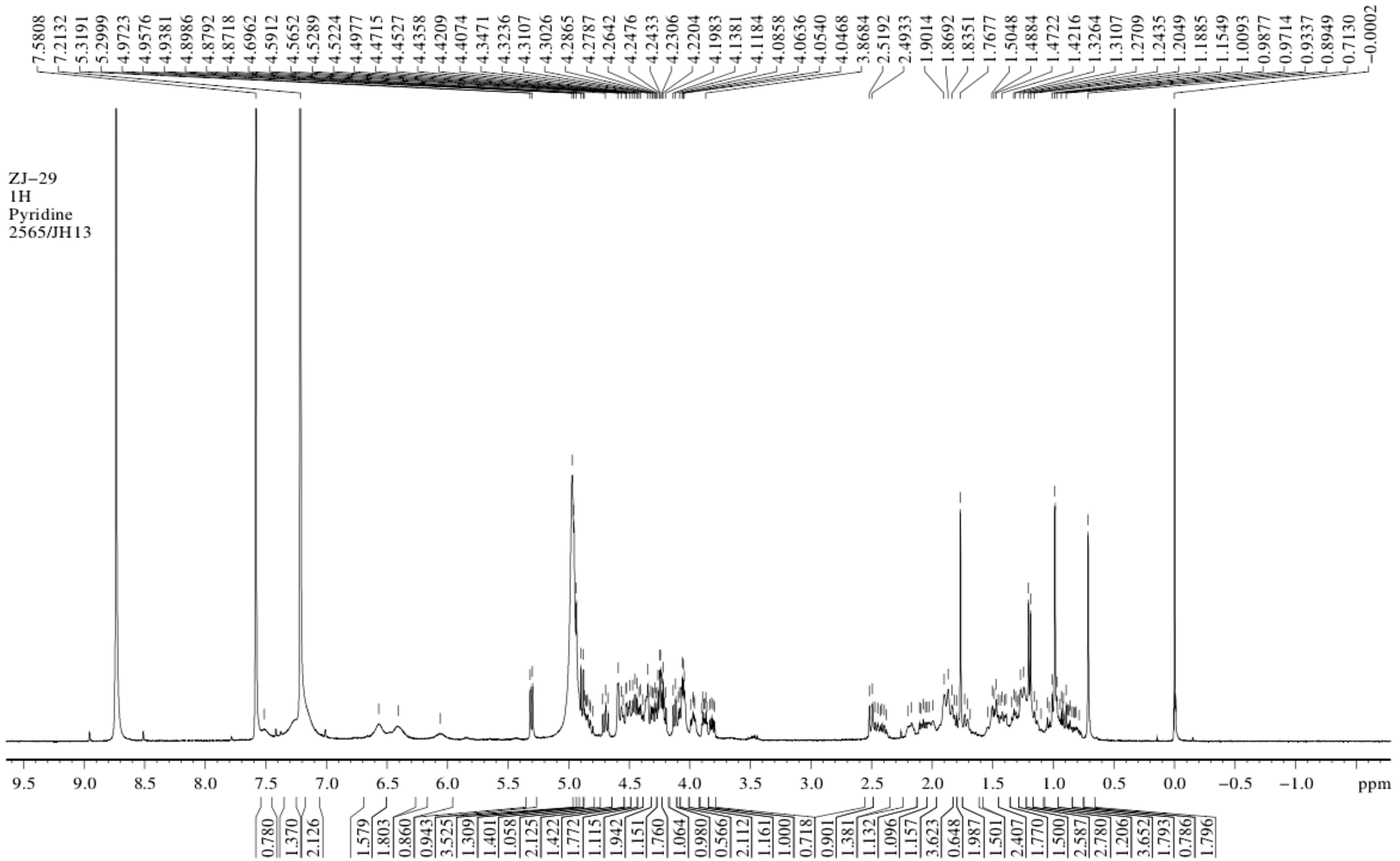

Figure S10. ^{1}H-NMR spectrum of compound **2**.

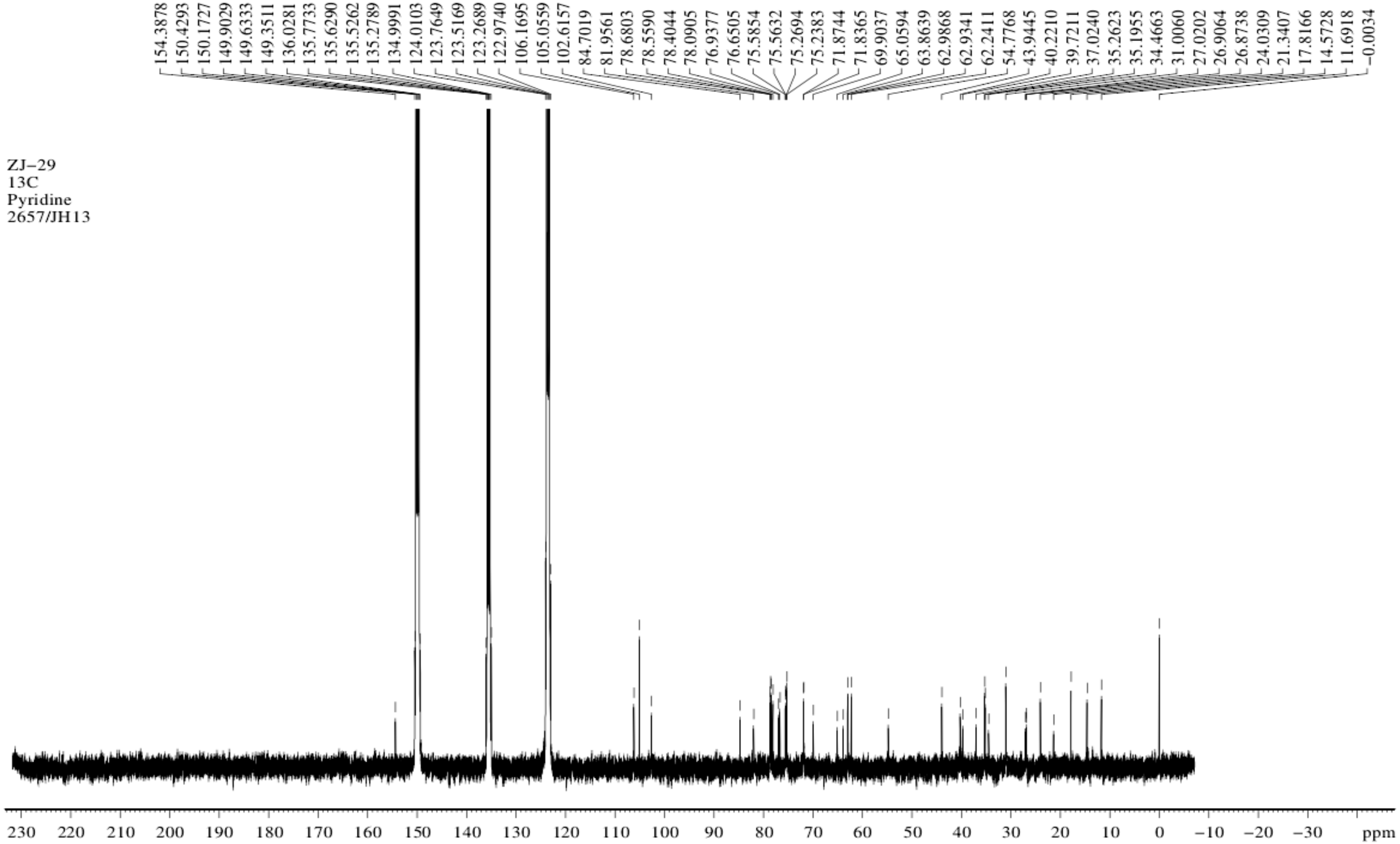

Figure S11. ^{13}C-NMR spectrum of compound **2**.

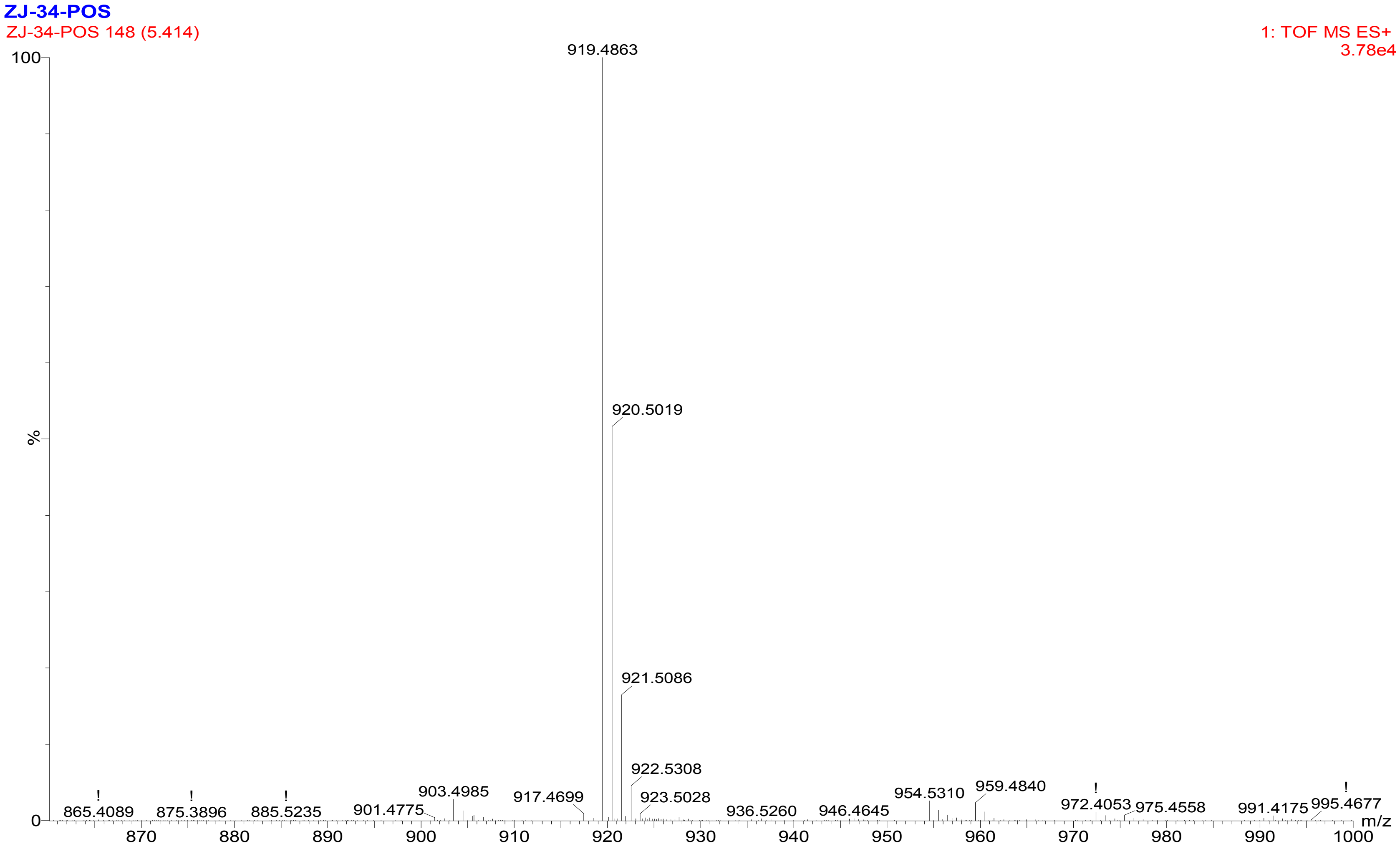

Figure S12. HR-ESI-MS spectrum of compound **3**.

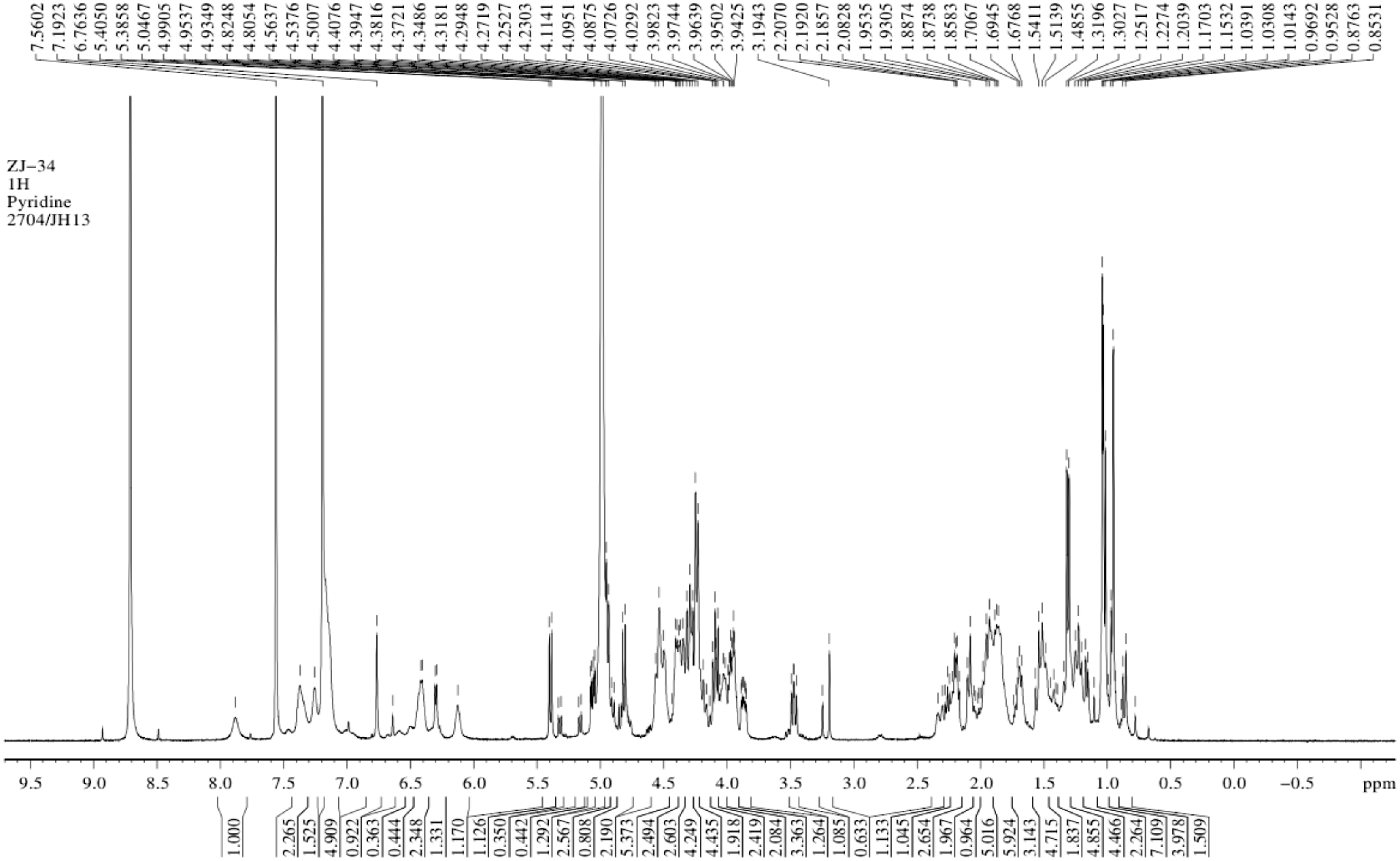

Figure S13. ^{1}H-NMR spectrum of compound **3**.

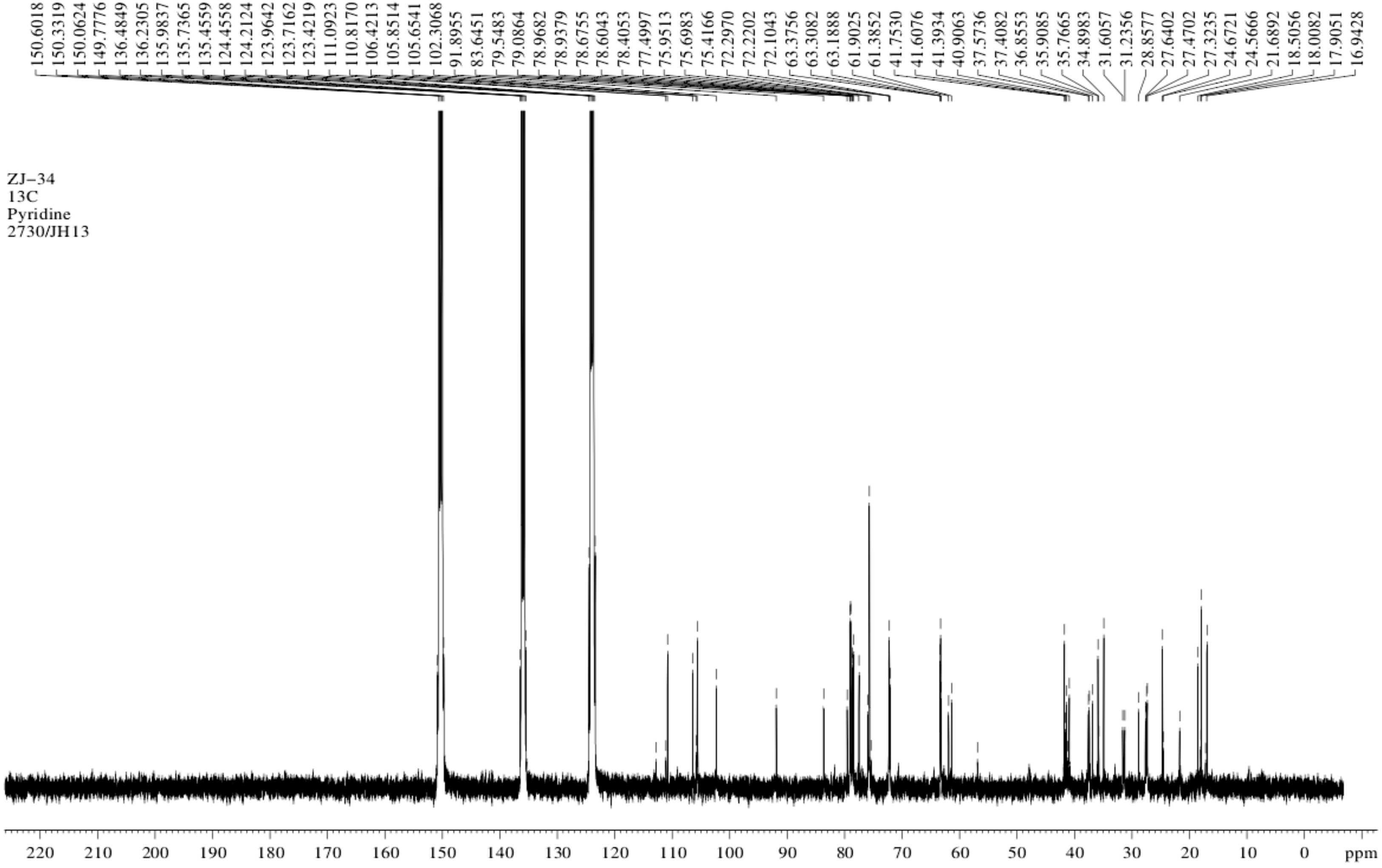

Figure S14. ^{13}C-NMR spectrum of compound **3**.

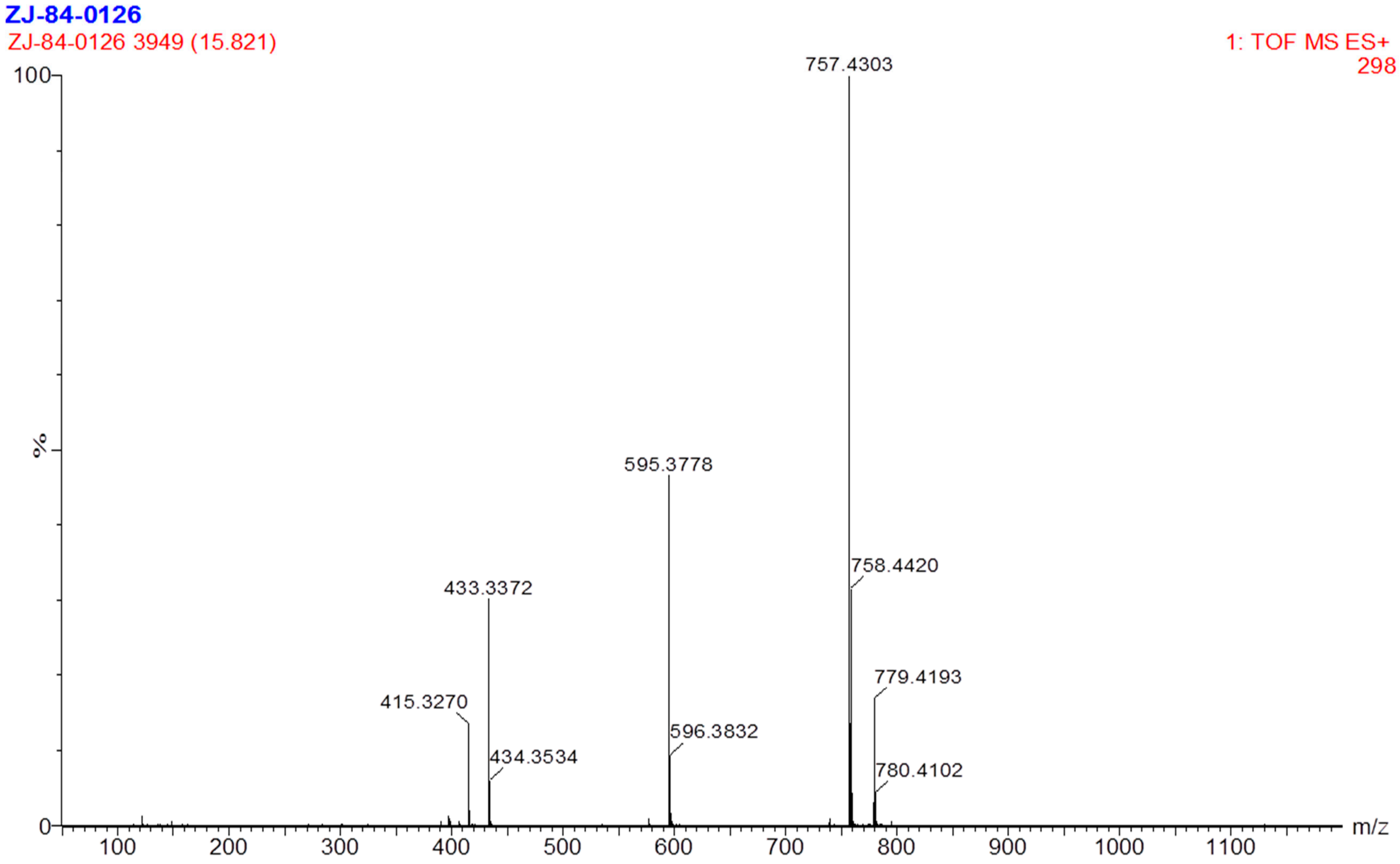

Figure S15. HR-ESI-MS spectrum of compound **4**.

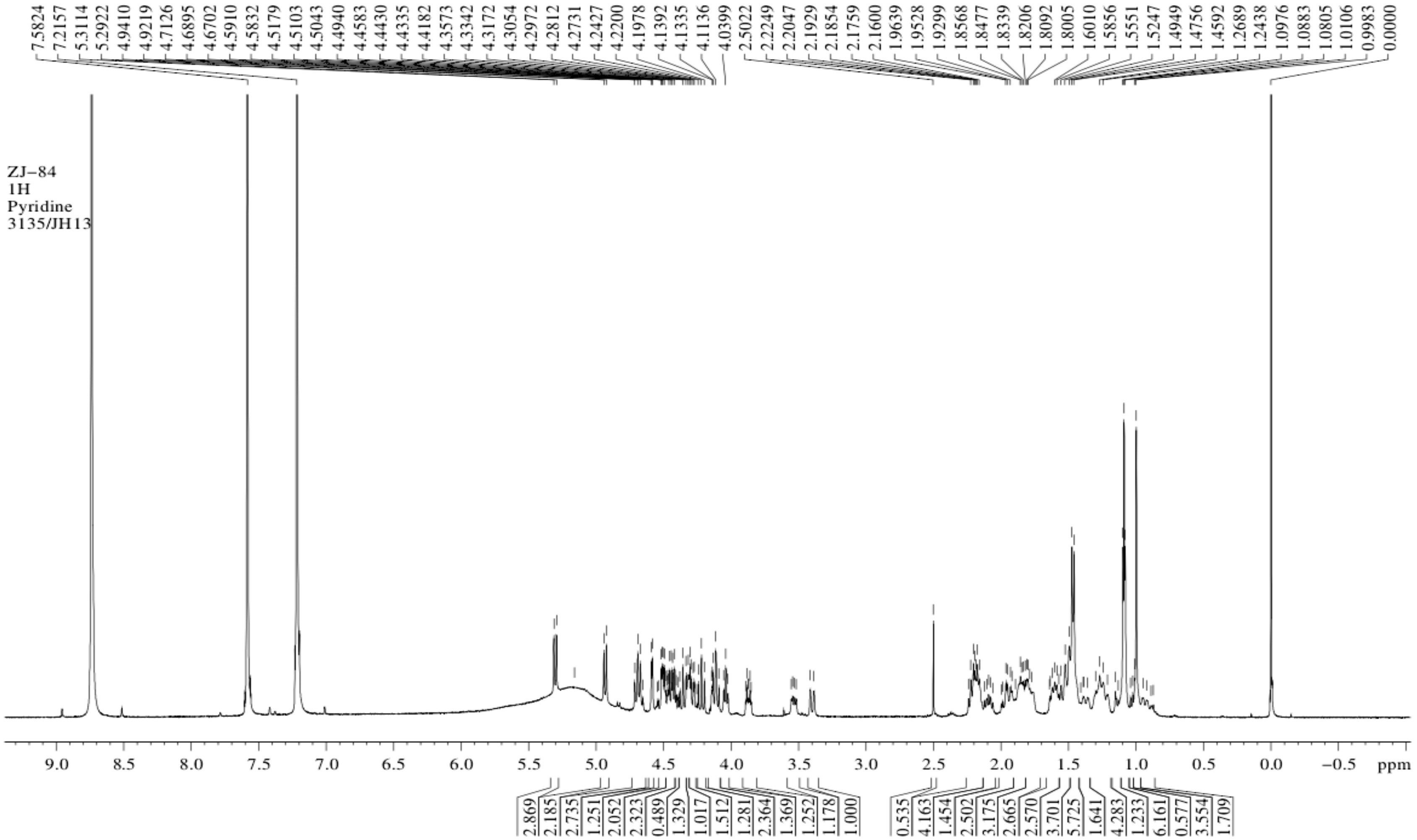

Figure S16. ^{1}H-NMR spectrum of compound **4**.

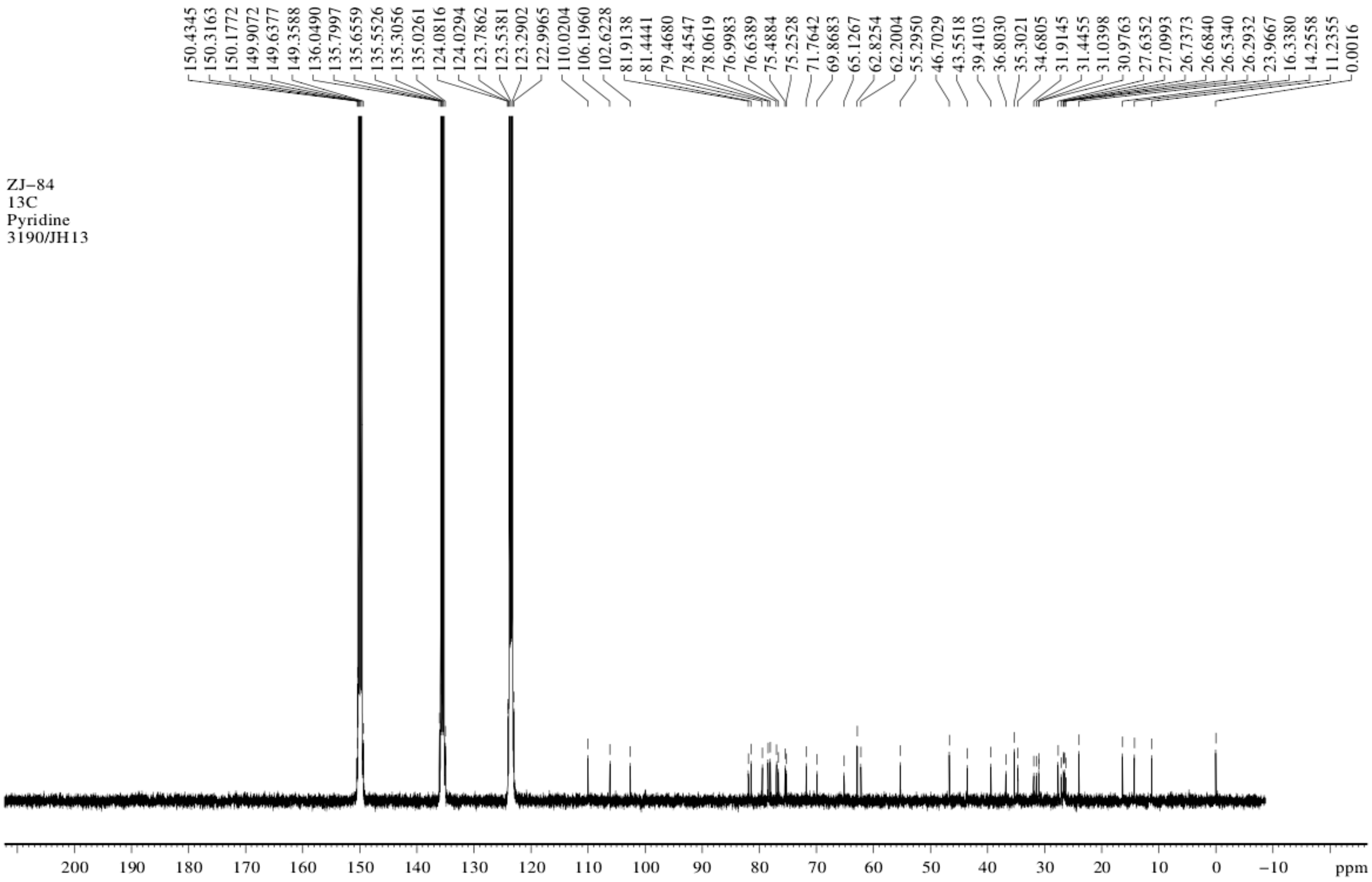

Figure S17. ^{13}C-NMR spectrum of compound **4**.
